# Auditory region circulation in Lagomorpha: the internal carotid artery pattern revisited

**DOI:** 10.1098/rstb.2022.0088

**Published:** 2023-07-03

**Authors:** Irina Ruf, Jin Meng, Łucja Fostowicz-Frelik

**Affiliations:** ^1^ Abteilung Messelforschung und Mammalogie, Senckenberg Forschungsinstitut und Naturmuseum Frankfurt, 60325 Frankfurt am Main, Germany; ^2^ Institut für Geowissenschaften, Goethe-Universität Frankfurt am Main, 60438 Frankfurt am Main, Germany; ^3^ Division of Paleontology, American Museum of Natural History, New York, NY 10024, USA; ^4^ Department of Organismal Biology and Anatomy, University of Chicago, Chicago, IL 60637, USA; ^5^ Department of Evolutionary Paleobiology, Institute of Paleobiology, Polish Academy of Sciences, 00-818 Warsaw, Poland

**Keywords:** internal carotid artery, Euarchontoglires, Leporidae, Ochotonidae, µCT scanning

## Abstract

The internal carotid artery (ICA) is one of the major vessels in the cranial circulation. Characters concerning the ICA, such as its course in the auditory region, have been employed frequently in phylogenetic analyses of mammals, including extinct taxa. In lagomorphs, however, our knowledge on vascular features of the auditory region has been based predominantly on living species, mostly on the European rabbit. We present the first survey on 11 out of 12 extant genera and key fossil taxa such as stem lagomorphs and early crown representatives (*Archaeolagus* and *Prolagus*). The ICA pattern shows a modified transpromontorial course in stem taxa (*Litolagus*, *Megalagus* and *Palaeolagus*) and *Archaeolagus*, which we propose as the ancestral character state for Lagomorpha, similar to that for the earliest rodents, plesiadapids and scandentians. The ICA pattern in leporids is perbullar, but shows structural similarities to stem taxa, whereas the extrabullar ICA course in *Ochotona* is apparently a highly derived condition. *Prolagus* shows a mixed character state between leporids and *Ochotona* in its ICA route. The persistence of the transpromontorial ICA course and similarities in the carotid canal structure among stem taxa and crown leporids support morphological conservatism in Lagomorpha, in contrast to their sister clade Rodentia.

This article is part of the theme issue ‘The mammalian skull: development, structure and function’.

## 1. Introduction

The paired carotid artery is the most important blood vessel supplying the head. It arises from the aortic arch as the common carotid artery, which branches into the external carotid artery (ECA), supplying most of the face and neck, and the internal carotid artery (ICA), which passing through the auditory region supplies the brain, orbit and nasal cavity through its offshoots: the anterior, middle and posterior cerebral arteries, ophthalmic artery, and ethmoidal artery, respectively [1–5]. However, the ICA itself does not contribute substantially to the blood supply of the ear region.

Characters concerning the organization and course of the internal carotid artery have been employed frequently in phylogenetic analyses of mammals, including extinct taxa [6–11], because the ICA foramina, canals or grooves are often preserved in skulls of fossil specimens.

The ancestral course of the ICA for Mammalia is accepted as medial to the promontorium of the petrosal [7,12], whereas for the Eutheria as lateral to the promontorium and in the tympanic cavity (see e.g. [12–14]). Interestingly, the reconstructed position of the ICA in the Late Cretaceous stem placentals *Asioryctes*, *Kennalestes* and *Zalambdalestes* is medial [10,12]. As currently understood, Matthew's [15] hypothesis suggesting the presence of two ICA branches (medial and lateral as postulated for several early eutherian taxa) is not valid, as the ICA at the auditory capsule's level originates from a single embryonic dorsal aorta independent from its later course [16].

In Eutheria, three main types of the ICA arrangement are observed, in its relation to the auditory region: (1) transpromontorial, where the ICA goes through the tympanic cavity and crosses the ventral surface of the promontorium of the petrosal; (2) perbullar, with the ICA contained within a canal in the medial wall of the auditory bulla; and (3) extrabullar, with the ICA passing the ear region externally, medial to the auditory bulla and the tympanic cavity [7]. The transpromontorial route of the ICA is recognized as an ancestral character state for Eutheria [14], whereas the perbullar and extrabullar courses of the ICA are considered derived conditions [7,10,11].

**Figure 1.**
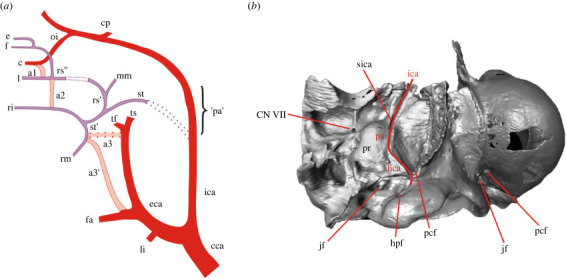
General circulation pattern of the carotid artery in extant Lagomorpha auditory region (*a*) and the ICA-related anatomic features in the fossil lagomorph *Palaeolagus* (*b*). a1, a2 (internal ophthalmic artery), a3, and a3′ (first part of mandibular artery), anastomoses; c, ciliary artery; cca, common carotid artery; cp, posterior communicating artery; e, ethmoidal artery; eca, external carotid artery; f, frontal artery; fa, facial artery; hpf, hypoglossal foramen; ica, internal carotid artery; jf, jugular foramen; l, lacrimal artery; li, lingual artery; lica, lateral internal artery; mm, medial meningeal artery; oi, ophthalmic artery; 'pa', promontarial artery; pcf, posterior carotid foramen; pr, promontorium; ri, infraorbital artery; rm, mandibular artery; rs' and rs'', distal parts of the supraorbital artery; sica, sulcus of internal artery; st, stapedial artery; st', distal part of stapedial artery; tf, transverse facial artery; ts, temporal superfacial artery; CN VII, entrance of CN VII (facial nerve). Carotid artery branches marked in red; stapedial artery and its offshoots marked in purple; anastomoses between the branches of the carotid artery and distal part of the stapedial artery marked in pink; the parts of the blood vessels partly obliterated are marked with dashed lines.

Thus far, our knowledge on vascular features of the auditory region in lagomorphs (figure 1), such as the ICA organization, has been based almost exclusively on living species and mostly on the European rabbit (*Oryctolagus cuniculus*; see [1,3,17,18]). Bugge [17,18] provided a detailed description of the cephalic arterial system, i.e. internal and external carotid arteries and stapedial artery including anastomoses, of extant Lagomorpha (three genera). While the internal carotid artery is relatively well developed in *O. cuniculus*, European hare (*Lepus europaeus*) and Afghan pika (*Ochotona rufescens*), the stapedial artery is substantially reduced (figure 1*a*). Its tympanic (proximal) part is obliterated and mostly lost, whereas the distal portion and its branches are supplied by the ECA via anastomoses (see [17;18, fig. 15D]). The two living lagomorph families, Ochotonidae and Leporidae, exhibit different character states of the ICA: the former, the extrabullar, while the latter, the perbullar condition [7,18].

The skull anatomy of several fossil lagomorph species has been described in detail (e.g. [19–22]), but only Wolniewicz & Fostowicz-Frelik [23] devoted more attention to the ICA-related features. Wu [24] marks the external opening of the carotid canal ‘external carotid foramen’ *sensu* [24] in the fossil ochotonid *Alloptox*, similar to the opening in *Prolagus sardus* shown in Dawson [21]; Meng *et al*. [25] describe the tympanic–petrosal complex in another fossil pika, tentatively assigned to *Sinolagomys*, in which the spongy bulla shows an external opening for the ICA. Other contributions concerning the auditory region in lagomorphs are scarce and focused mostly on the organs of hearing (see [26–28]). Thus, our knowledge on the cranial circulation in the ear region of extinct (and most extant) lagomorphs is extremely limited.

Here we provide the first comprehensive and µCT data-based morphological survey of the ICA-related structures traceable in the skull of Lagomorpha. Our study includes all but one of the extant lagomorph genera and a few fossil taxa for which the course of the internal carotid artery through the ear region could be ascertained from osteological features. This paper furthers the view of lagomorphs as an emergent robust system (see [29]) in which we can study macro- and micro-scale patterns of morphological change.

## 2. Material and methods

The auditory region of seven fossil and 29 extant lagomorph specimens has been studied, using the µCT scans and virtual three-dimensional reconstructions performed in Avizo 9.0.1 (Thermo Fisher Scientific 1995–2019) and VG Studio MAX2.2 (Volume Graphics, Heidelberg, Germany) software. The sample of fossil Lagomorpha comprises North American stem taxa: *Megalagus turgidus* (FMNH UC/PM 1642, Field Museum of Natural History, Chicago, IL) from the early Oligocene of Nebraska, *Litolagus molidens* (AMNH FM 143955, American Museum of Natural History, New York, NY) from the earliest Oligocene of Wyoming and two *Palaeolagus* species (Oligocene *Palaeolagus* *burkei* AMNH 8709 and early Oligocene *Palaeolagus* *haydeni* FMNH PM 9476), as well as the leporid *Archaeolagus ennisianus* (AMNH FM 7190) from the earliest Miocene of Oregon, and the extinct Pleistocene/Holocene ochotonid *Prolagus sardus* (AMNH 116812) from Sardinia, Italy. The osteological material of all specimens apart from *Prolagus* and *Archaeolagus* is represented by almost complete skulls (for details, see [22,23,30]). The holotype skull of *A. ennisianus* is partially preserved with one auditory bulla present, whereas the studied specimen of *P. sardus* is an isolated petrosal associated with the ectotympanic bulla.

Extant Leporidae are represented by cleaned skulls of 24 species comprising all genera except for the monospecific *Bunolagus*. Ochotonidae include cleaned skulls of five extant *Ochotona* species (*Ochotona* *alpina*, *Ochotona* *collaris*, *Ochotona* *dauurica*, *Ochotona* *rutila* and *Ochotona* *thibetana*). We studied both ear regions (at the left and right sides of a skull) in each specimen if available. Detailed information on the species sample and scan parameters are given in electronic supplementary material, table S1. The visual data including three-dimensional models of the region of interest are provided in the Dryad Digital Repository [31].

## 3. Comparative morphology

### 3.1. Stem Lagomorpha

**Figure 2.**
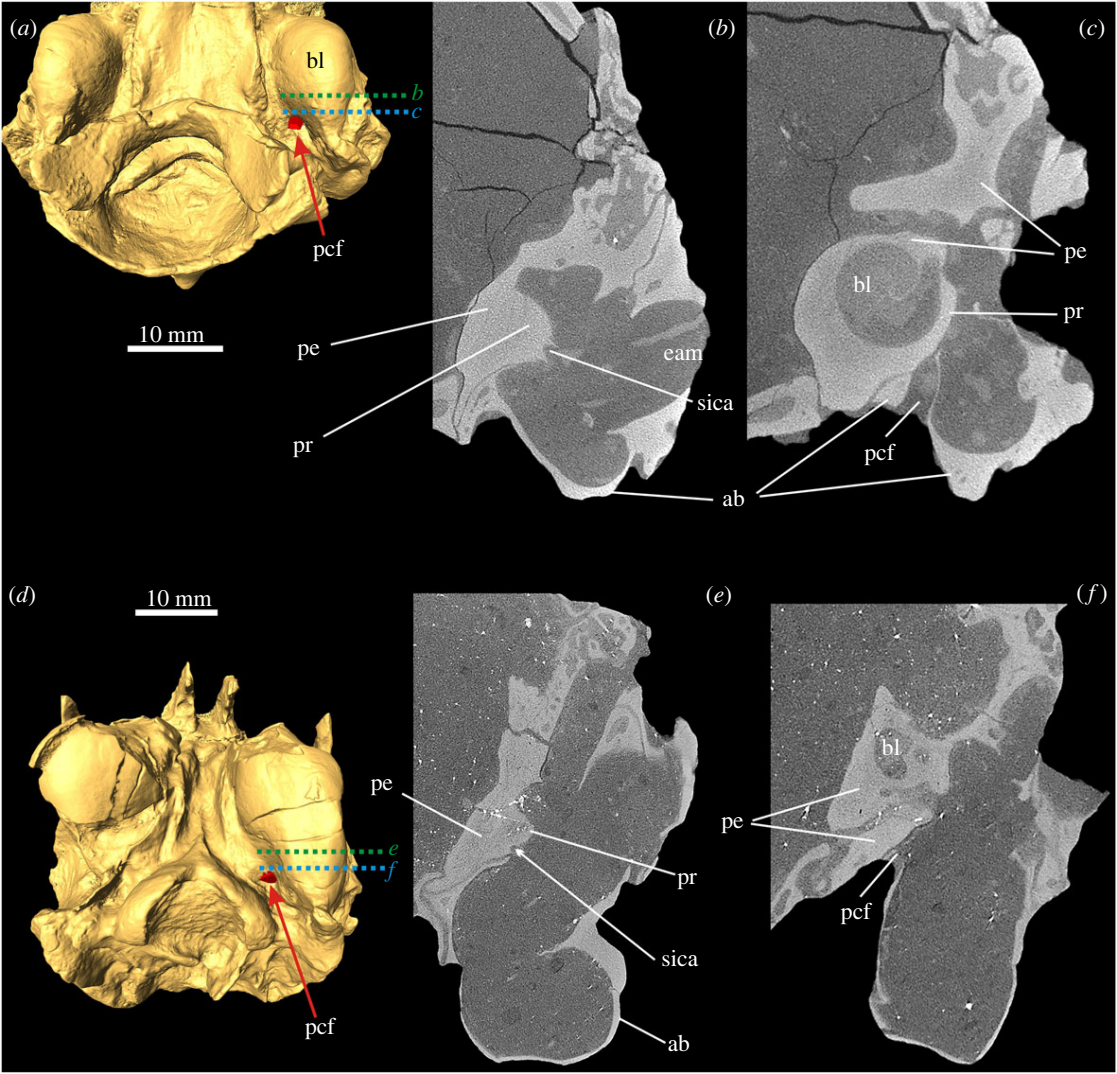
Auditory region of lagomorphs from the Eocene–Oligocene transition period. (*a*–*c*) *Megalagus turgidus* (FMNH UC 1642), Brule Formation, Grime's Ranch, Sioux County, Nebraska, USA. (*d*–*f*) *Litolagus molidens* (AMNH FM 143955), Herman Wulff Ranch, Converse County, Wyoming, USA. (*a*,*d*) Three-dimensional models of the ear region in ventral view, internal carotid artery (ica) marked in red (see arrow); the sections shown in (*b*), (*c*), (*e*) and (*f*) are marked as dotted lines. (*b*,*c*) transversal µCT images of the right ear (mirror image to enable comparisons with (*e*,*f*)) from anterior (*b*) (green section line in (*a*)) to posterior (*c*) (blue section line in (*a*)). (*e*,*f*) Transversal µCT images of the left ear from anterior (*e*) (green section line in (*d*)) to posterior (*f*) (blue section line in (*d*)). ab, auditory bulla (ectotympanic); bl, bony labyrinth; eam, external auditory meatus; pcf, posterior carotid foramen; pe, petrosal; pr, promontorium; sica, sulcus for internal carotid artery.

In *Megalagus turgidus*, the posterior carotid foramen (PCF, the external opening into the carotid canal) enters the bulla in its posteromedial corner, directly anteromedial to the jugular foramen (figure 2*a–c*). The entrance is completely formed by the ectotympanic. In its course, the short proximal (or posterior) carotid canal is formed by the ectotympanic and petrosal and continues as a distinct and deep sulcus crossing the medioventral surface of the promontorium in a lateral curve. Anteriorly, it leaves the tympanic cavity in the medial direction; this area is not well preserved in the specimen.

**Figure 3.**
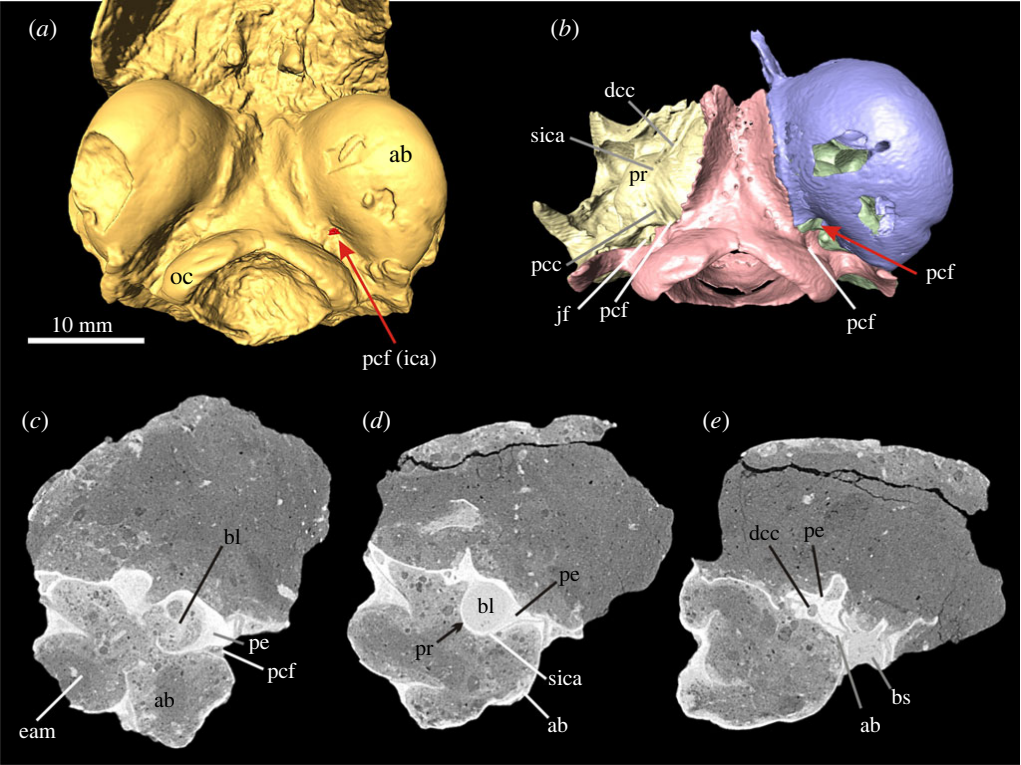
Early lagomorph *Palaeolagus haydeni* (FMNH PM 9476), early Oligocene of the Brule Formation, Nebraska, USA. (*a*) Three-dimensional model of ear region in ventral view, entrance of the internal carotid artery marked in red (see arrow); (*b*) ear region in ventral view, right auditory bulla removed ([23], modified). (*c*–*e*) *Palaeolagus burkei* (AMNH 8709), early Oligocene, Colorado, USA; transversal µCT images of the left ear from posterior (*c*) to anterior (*e*). For anatomical abbreviations, see captions to figures 1 and 2. bs, basisphenoid; dcc, distal carotid canal; oc, occipital condyle; pcc, proximal carotid canal.

In *Palaeolagus haydeni* and *Palaeolagus* *burkei*, the ICA enters the basicranium via the PCF, which lies between the ectotympanic and petrosal right in front of the jugular foramen (figure 3). The foramen opens into a relatively short proximal carotid canal that runs anterodorsally between these two bones and opens into the tympanic cavity. The ICA continues in a shallow sulcus on the medial side of the promontorium (figure 1*b*) and then becomes enclosed again, between the bulla and petrosal bone into the distal (anterior) carotid canal. The ICA then bends medially and enters the brain cavity next to the sella turcica. The anterior course of the ICA from the distal carotid canal into the brain cavity could not be fully traced in *P*. *burkei* owing to poor preservation in that area. The promontorium of both specimens is smooth and no additional sulci were detected.

In *Litolagus molidens* (figure 2*d*–*f*), the PCF leads into a short proximal carotid canal between the ectotympanic and petrosal. The posterior rim of the foramen is embraced by a small lamella of the bulla. The course of the ICA across the promontorium cannot be traced completely. Posteriorly, no sulcus is present, though it might be obscured by scan artefacts. Anteriorly, the sulcus for the ICA continues into a distal carotid canal formed by the petrosal and ectotympanic.

The whole course of the ICA through the ear region in stem Lagomorpha can be therefore divided into three distinct sections: (1) the posterior carotid foramen leading into a short proximal carotid canal (housing the posterior ICA section) between the ectotympanic and petrosal, (2) the middle (promontorial) section of the ICA, which runs in a sulcus at the ventromedial side of the promontorium, and (3) a long anterior section of the ICA, which is completely enclosed by the surrounding bones (especially the petrosal and ectotympanic) forming a distal (or anterior) carotid canal, before it enters the cranial cavity. *Megalagus* and *Litolagus* depart in certain respects from this scheme; in both species the posterior carotid foramen is totally or at least mostly located in the tympanic bulla, and in *Litolagus* the posterior part of the ICA promontorial section is not represented by a sulcus. In all cases, there is no evidence of the stapedial artery as a branch of the ICA, a known fact for the extant taxa [18]. However, it should be noted that the absence of specific osteological characters (e.g. sulci) may not, strictly speaking, attest to the absence of the respective blood vessels (see [8]).

### 3.2. Crown Lagomorpha

**Figure 4.**
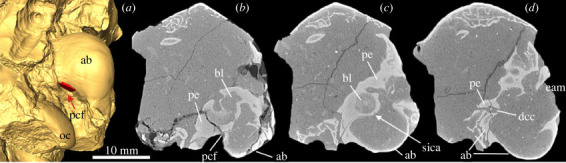
Auditory region of the archaeolagine *Archaeolagus ennisianus* (AMNH FM 7190), Miocene of the John Day Formation, north fork of John Day River, Oregon, USA. (*a*) Three-dimensional model of the left ear region in ventral view, entrance of internal carotid artery (ica) marked in red (see arrow); (*b–d*) transversal µCT images of the left ear from posterior (*b*) to anterior (*d*). For anatomical abbreviations, see captions to figures 1–3.

The early archaeolagine leporid *Archaeolagus ennisianus* shows a huge PCF located in front of the jugular foramen between the ectotympanic and petrosal (figure 4). The carotid canal and the course of the ICA across the promontorium resemble the pattern observed in the stem lagomorphs under study, i.e. there is a shallow sulcus formed in the tympanic surface of the petrosal. The skull of *A. ennisianus* is only partially preserved; thus, the anteriormost course of the ICA into the cranial cavity is not fully recognizable.

**Figure 5.**
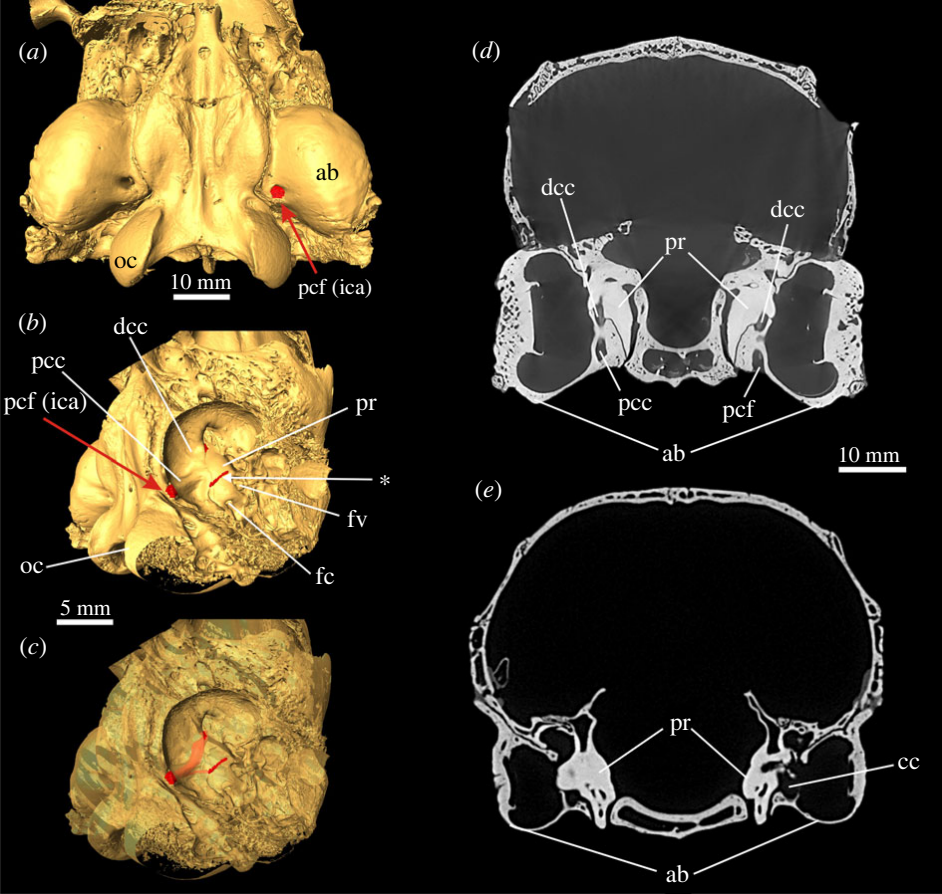
Auditory region and position of the entrance of the internal carotid artery in extant leporids. (*a–d*) *Oryctolagus cuniculus* (M4860). (*a*) Three-dimensional model of the ear region in ventral view, anterior to the top, entrance of internal carotid artery in red (see arrow); (*b*) the same specimen in left lateral view with auditory bulla removed, asterisk (*) indicates branch of the internal carotid nerves (?) running in a shallow sulcus of the promontorium; (*c*) as in (*b*), but bones made transparent; (*d*) transversal µCT image of the ear region of the same specimen; (*e*) transversal µCT image of the ear region of *Pentalagus furnessi* (M12940), the carotid canal is entirely situated between the ectotympanic and petrosal. For abbreviations, see captions to figures 1–3. cc, carotid canal; fc, fenestra cochleae; fv, fenestra vestibuli.

All studied extant leporid specimens have a similar pattern of the osteological features related to the perbullar course of the ICA in the ear region. The auditory bulla shows a distinct PCF (for the ICA entrance) that is entirely formed by the ectotympanic and located posteromedially to medially in the bulla (figure 5), in the vicinity of the jugular foramen. The PCF opens into a wide carotid canal for the ICA. The proximal (posterior) part of the canal is made up by the ectotympanic only (apart from *Nesolagus timminsi* and *Pentalagus furnessi*), whereas the distal (anterior) part runs anterodorsally between the ectotympanic and the petrosal. Along its course, the carotid canal does not enter the tympanic cavity, in contrast to the fossil taxa under study. However, before it bends medially toward the brain cavity (area of the sella turcica), its lateral wall can be incomplete and, thus, the canal lumen becomes confluent with the anteriormost part of the tympanic cavity in the area of the foramen lacerum medium. Most studied specimens have no distinct sulci on the promontorium for e.g. branches of the tympanic plexus; however, some species show certain specific characters due to the exact position of the PCF and the course of the carotid canal, which can vary even within a genus. In *Caprolagus hispidus*, *Lepus* spp., *Pronolagus* spp. and *Sylvilagus* spp., the carotid canal is situated anterior to the bony labyrinth (and the promontorium) and thus shows a steeper course. *Pronolagus* cf. *saundersiae* has a smaller foramen (possibly for the internal carotid nerves) directly in front of a large foramen; we interpret the latter as the PCF. Both canals are confluent inside the ectotympanic. In *Oryctolagus cuniculus* and *C. hispidus*, a very small canal branches off the proximal part of the carotid canal and runs anterodorsally between the ectotympanic and petrosal; more anteriorly, it enters the tympanic cavity and continues into a shallow sulcus on the promontorium that runs anteriorly and medially to the fenestra vestibuli (figure 5). This canal and sulcus may house a branch of the internal carotid nerves (internal carotid plexus). Krause [1] described a distinct tympanic sulcus on the promontorium of *O. cuniculus* that houses the tympanic nerve (branch of CN IX); however, our structure seems to be located more anteriorly and thus does not refer to the tympanic nerve as it is clearly associated with the carotid canal. *Lepus townsendii*, *Pronolagus rupestris* and *Sylvilagus nuttallii* also show the small additional canal but no sulcus on the promontorium. In *Lepus sinensis* and *Sylvilagus brasiliensis*, a tiny separate canal is present posterior to the carotid canal that shows the same course (including a shallow sulcus on the promontorium) and corresponds to the small canal observed in *Oryctolagus* and *Caprolagus*. *Sylvilagus floridanus* has two small canals branching off from the carotid canal; the anterior one leads into a sulcus on the anterior tip of the promontorium.

*Pentalagus furnessi* and *Nesolagus* spp. have very small auditory bullae compared with those of most other leporids; they also differ in the PCF location and morphology. In *Pentalagus*, the PCF lies just at the posteromedial rim of the ectotympanic. The foramen is not fully formed by the ectotympanic but in part also by the petrosal. The canal is quite short and is entirely made up by the ectotympanic and petrosal. *Nesolagus netscheri* and *N. timminsi* show two foramina in the region of interest. A large foramen, most probably the PCF, enters the auditory region right in front of the jugular foramen. In *N*. *netscheri*, this foramen lies within the ectotympanic; in *N*. *timminsi*, contrary to other leporids, it is clearly situated between the auditory bulla and petrosal. In both species, the foramen opens into a short carotid canal that runs entirely between the two bones (ectotympanic and petrosal), similar to *Pentalagus*. However, a smaller canal that is confluent with the former is totally enclosed by the ectotympanic and its entrance is situated anteromedially to the large foramen. The promontorium in both *Nesolagus* species shows no sulci connected to the carotid canal.

**Figure 6.**
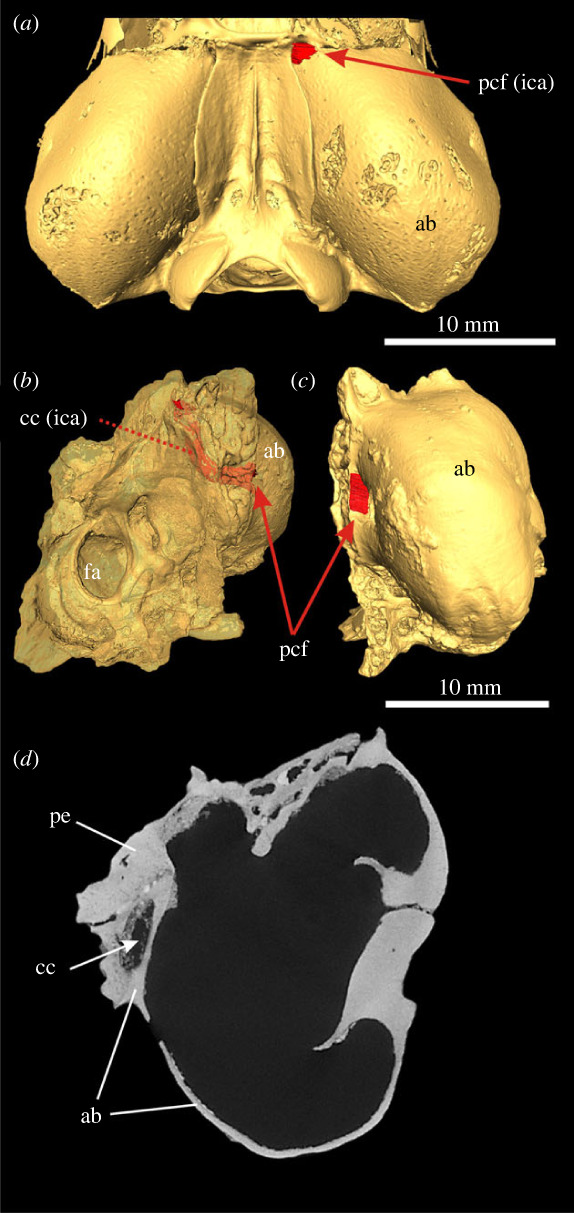
Position of internal carotid artery in Ochotonidae. (*a*) Three-dimensional model of the ear region of *Ochotona alpina* (M100488) in ventral view, position of the entrance of internal carotid artery in red (see arrow). (*b–d*) *Prolagus sardus* (AMNH116812). (*c*) Three-dimensional model of the right ear region (mirror image) in ventral view, anterior to the top, entrance of internal carotid artery in red (see arrow); (*b*) transparent three-dimensional model of the same specimen in oblique anteromedial view (mirror view), anterior to the left; the course of internal carotid artery in carotid canal indicated in red (see arrow and dotted line); (*d*) transversal µCT image of the same specimen (mirrored). For anatomical abbreviations, see captions to figures 1 and 2.

In extant ochotonids, the ICA enters the cranial cavity (figure 6*a*) via the foramen lacerum medium (carotid notch of the piriform fenestra according to [32]). According to Bugge [18], the ICA runs below the medial tympanic bulla in an anterior direction before entering the basicranium. Absence of a distinct intraosseous course of the ICA in the auditory region, and thus an extrabullar course of the ICA, is confirmed in all the pika species in our study.

Dawson [21] described the external cranial anatomy of the fossil ochotonid *Prolagus sardus* and observed the PCF located anteromedially in the bulla (see her fig. 4). This is confirmed by our studied specimen of *P. sardus* (AMNH 116812). A short carotid canal is traceable in the anterior auditory region that runs from ventral to dorsal into the cranial cavity (figure 6*b–d*). The PCF is formed by the ectotympanic and opens into the anteromedial part of the auditory bulla. Anteriorly, the carotid canal is formed by the ectotympanic and petrosal. The canal does not enter the tympanic cavity. Unfortunately, our specimen shows an incomplete promontorium and posterior auditory bulla; thus, no further information on additional small canals and sulci is available.

## 4. Discussion

In a broader phylogenetic scheme of the Euarchontoglires clade, lagomorphs noticeably differ in the course of the ICA from rodents, their sister clade within the Glires cohort [8]. Rodents show a great diversity of the ICA course patterns and of related structures (see [18,33]); they display all three types of the ICA route—transpromontorial, perbullar and extrabullar [12]—early in their evolution. Some most basal taxa, such as *Cocomys*, *Sciuravus* and *Paramys* show a transpromontorial course of the ICA [8], although *Reithroparamys*, a more specialized ischyromyid rodent already displays complete lack of the ICA (see [8,34]). The ancestral state for Glires was presumably the transpromontorial course of the ICA as this character state is shared by stem Lagomorpha with the primatomorph *Plesiadapis* ([35]; see [36] for phylogenetic position) and above-mentioned earliest rodents (ctenodactyloids, ischyromyids and sciuravids). The exact spatial relationships between the promontorium and ICA course within the tympanic cavity are, however, not the same in stem Lagomorpha as in primatomorphs and other Euarchontoglires, which have the transpromontorial ICA course. Furthermore, the ICA (especially its promontorial part) in stem lagomorphs is positioned differently from the reconstructed ICA morphology in early eutherians (see [7,13,35]). In lagomorphs, the sulcus for the ICA on the promontorium is more medial (especially in *Palaeolagus* and *Litolagus*) than in other Euarchontoglires displaying this character state.

The transpromontorial course of the ICA is observed in some Euarchontoglires groups, such as treeshrews (*Ptilocercus* and Tupaiidae; see [37]), Strepsirhini, Paromomyidae and Plagiomenidae (see [12]), but in all these groups the course of the ICA runs mostly in the middle of the promontorium. In Dermoptera the ICA is lost, although the presence of the internal carotid nerves indicates a previous transpromontorial course in a similar position (see fig. 6 in [35]). Therefore, the ICA course in stem Lagomorpha cannot be interpreted strictly as ancestral compared with the totality of Euarchontoglires. Its position is already derived, although, being still transpromontorial, it should be regarded as a modified character state in relation to the primitive course known for other Euarchontoglires. A somewhat similar situation occurs in *Tupaia*, in which a bony canal encloses the ICA, but still within the tympanic cavity, and thus it is a derived state compared with an open transpromontorial sulcus [8,35].

Nevertheless, the transpromontorial course of the ICA and the presence of a sulcus in the Oligocene stem lagomorphs and especially early Miocene *Archaeolagus* are yet further confirmation of the notable morphological conservatism observed in Lagomorpha (see [18,23,30,38]).

The only other derived feature in stem Lagomorpha seems to be a lack of the stapedial artery originally existing in their placental ancestors [8]. However, the stapedial foramen, normally passed by the stapedial artery, is retained as a plesiomorphic mammalian character in Lagomorpha (see [39]). The uniform pattern of the ICA route in stem lagomorphs stands in opposition to the rapid evolutionary changes observed in rodents and even advanced eurymylid Glires, such as *Rhombomylus*, which has already lost the PCF and a whole ICA, whereas in the closely related *Matutinia* the ICA presumably showed an extratympanic bifurcation of the ICA into a branch representing the anterior continuation of this vessel and the stapedial artery, with separate entrances [8,40].

**Figure 7.**
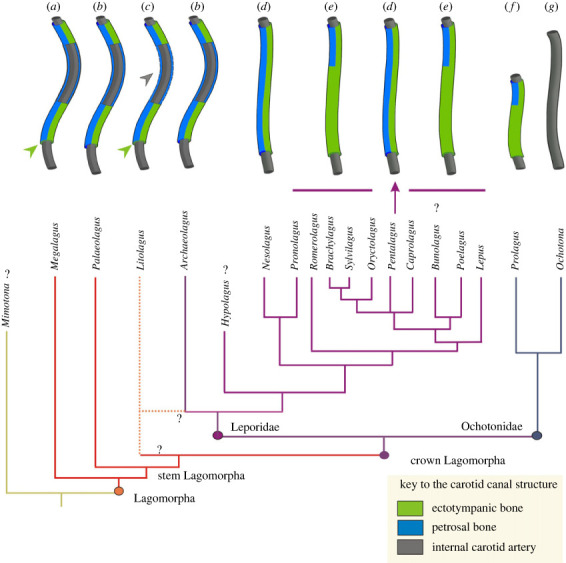
The carotid canal structure in Lagomorpha. Schematic drawings in ventral view, anterior to the top. (*a*) Posterior and anterior carotid canals present, the posterior carotid foramen formed exclusively by ectotympanic (green arrow), complete sulcus on the promontorium; (*b*) posterior and anterior carotid canals present, the posterior carotid foramen formed by ectotympanic and petrosal, complete sulcus on the promontorium; (*c*) posterior and anterior carotid canals present, the posterior carotid foramen formed mostly by ectotympanic (green arrow), incomplete sulcus on the promontorium (grey arrow); (*d*) complete carotid canal with the posterior carotid foramen formed by ectotympanic and petrosal; (*e*) complete carotid canal formed mostly of the ectotympanic; (*f*) short version of (*e*); (*g*) extrabullar carotid canal. Lagomorph phylogeny based on Matthee *et al*. [41], Fostowicz-Frelik [22], Fostowicz-Frelik & Meng [42] and Cano-Sánchez *et al*. [43], modified. The dotted lines denote alternative phylogenetic position.

There is a disparity in the ICA course and the carotid canal structure among the stem and crown Lagomorpha (figure 7) as well as within the crown groups themselves (table 1 for details). In general, all studied stem fossil taxa and *Archaeolagus* allowed the reconstruction of a more or less medial transpromontorial course of the ICA. The extrabullar ICA course of the extant *Ochotona* is apparently highly derived, whereas the ICA course in extant Leporidae shows principally a similar pattern to that observed in stem lagomorphs (and *Archaeolagus*). However, both groups differ in the formation of the PCF, lack of the promontorial sulcus and total enclosure of the carotid canal, especially by the bullar wall.

**Table 1.** The structure of the posterior carotid foramen (PCF) and the carotid canal composition in fossil and extant Lagomorpha.

taxon	ICA course	PCF composition	proximal carotid canal composition	distal carotid canal composition	sulcus on the promontorium between proximal and distal carotid canal
*Megalagus turgidus*	transpromontorial	ectotympanic	ectotympanic + petrosal	ectotympanic + petrosal	complete
*Palaeolagus haydeni*	transpromontorial	ectotympanic + petrosal	ectotympanic + petrosal	ectotympanic + petrosal	complete
*Palaeolagus burkei*	transpromontorial	ectotympanic + petrosal	ectotympanic + petrosal	ectotympanic + petrosal	complete
*Litolagus molidens*	transpromontorial	ectotympanic + petrosal)	ectotympanic + petrosal	ectotympanic + petrosal	incomplete
*Archaeolagus ennisianus*	transpromontorial	ectotympanic + petrosal	ectotympanic + petrosal	ectotympanic + petrosal	complete
*Prolagus sardus*	perbullar	ectotympanic	ectotympanic	ectotympanic + petrosal	—
extant Leporidae	perbullar	ectotympanic^a^	ectotympanic^b^	ectotympanic + petrosal	—
extant Ochotonidae (*Ochotona*)	extrabullar	—	—	—	—

^a^In *Nesolagus timminsi*, the posterior carotid foramen is formed by both the ectotympanic and petrosal. This is also the case in *Pentalagus furnessi* (right side).

^b^In *Nesolagus* spp. and *P. furnessi*, a short carotid canal is completely formed by the ectotympanic and petrosal.

Among extant leporids, *Nesolagus* and *Pentalagus* are exceptions having the posterior part of the carotid canal and PCF formed also by both the ectotympanic and petrosal (figure 7), in this respect slightly resembling the ICA structure in stem lagomorphs (disregarding the absence of a transpromontorial sulcus). It is hard to discern whether such similarity is a result of a preserved ancestral structure, or a secondary derived character state, and its adaptive meaning is also unclear. The other feature of the ear structure that both extant genera (*Nesolagus* and *Pentalagus*) have in common with *Megalagus* is a relatively small auditory bulla. On the other hand, *Litolagus* and *Palaeolagus*, which also have the carotid canal built by both the ectotympanic and petrosal, display large auditory bullae. The size of the bullae in lagomorphs is regarded as an adaptive character related to the habitat type [22]; the species living in open landscape habitats or mixed forest/grassland environments have larger bullae than typical forest/jungle inhabitants (such as *Nesolagus* and *Pentalagus*). In rodents, bullar hypertrophy is frequently associated with increased aridity and xeric habitats (see [44]).

As there is no direct functional relation between the carotid canal structure and the size of the auditory bullae, we can assume that the ICA-related structures such as the PCF and presence/absence of the canal, and its composition, can be perceived as features of phylogenetic meaning in Lagomorpha. Although our study does not cover many Archaeolaginae and early Leporinae, it captures the ICA route and structure in most crucial points of the lagomorph evolution: stem taxa, early crown members and all two/three crown groups (depending on whether or not *Prolagus* is placed within Ochotonidae).

The character polarization is clear, and it follows the character states observed by Wible [12] for Placentalia: the transpromontorial course of the ICA with a well-displayed sulcus is an ancestral state for Lagomorpha, whereas the perbullar and extrabullar states should be considered as derived.

Furthermore, the PCF formed in parts by the ectotympanic and petrosal represents another ancestral character state for Lagomorpha, whereas the PCF formed solely by the ectotympanic can be considered derived. We can hypothesize that the carotid canal formed equally by the ectotympanic and petrosal (as in *Nesolagus* and *Pentalagus*) is less advanced than that formed prevailingly by the ectotympanic (the rest of the studied leporids). Following this hypothesis, it can be assumed that *Nesolagus* and *Pentalagus* are relatively ‘basal’ leporid taxa, which at least partly agrees with the recent molecular-based phylogenies of extant Lagomorpha (see e.g. [43]). Nevertheless, we cannot rule out that this is a secondary character because evolutionary parallelisms are frequent in lagomorph evolution [38,42]. The question remains unanswered until a more exhaustive study of the Neogene leporids is conducted. However, the persistence of the ‘stem lagomorph’ pattern of the ICA-related structures in *Archaeolagus* shows the evolutionary stability of this structure. The developmental aspect supports our conclusions as the pattern observed in the stem lagomorphs resembles the one described in prenatal ontogeny of *Oryctolagus cuniculus* [45]. In the European rabbit, the proximal part of the ICA becomes enclosed by the growing ectotympanic, and the distal part runs in a sulcus on the anterior promontorium but becomes enclosed by the ectotympanic and petrosal later.

In terms of the general ICA pattern found in Leporidae, it can be treated as an advanced variant of the basic stem lagomorph structure, especially when the fully extrabullar ICA course of modern ochotonids is considered (figure 7). In this respect, the pattern displayed by *Prolagus sardus* seems halfway between the typical leporid and ochotonid structures, as it is extrabullar in its posterior part but then becomes perbullar and the canal structure is similar to that of leporids (figure 7). Furthermore, the PCF is shifted far anteriorly and thus resembles a transitional stage. The *Prolagus* lineage is old, originating around the end of the Oligocene [46]. Whether *Prolagus* indeed shares a direct common ancestor with *Ochotona* is another matter, but the ICA structure and canal pattern show some similarity to the leporid structure, which suggests an independent evolutionary history of the *Prolagus* lineage from the rest of Ochotonidae. Until more fossil ochotonids are studied, it is hard to determine if the *Prolagus* pattern is ancestral for the entire ochotonid lineage and the fully extrabullar condition appeared later in the *Ochotona* lineage, or *Prolagus* is an independent group of the crown Lagomorpha.

## Data Availability

Virtual reconstructions of the auditory region of six fossil and two extant lagomorphs are available from the Dryad Digital Repository: doi:10.5061/dryad.prr4xgxrd [31]. Detailed data for all specimens, including µ-CT scan parameters, are provided in electronic supplementary material, table S1.
